# The Video Head Impulse Test to Assess the Efficacy of Vestibular Implants in Humans

**DOI:** 10.3389/fneur.2017.00600

**Published:** 2017-11-14

**Authors:** Nils Guinand, Raymond Van de Berg, Samuel Cavuscens, Maurizio Ranieri, Erich Schneider, Floor Lucieer, Herman Kingma, Jean-Philippe Guyot, Angélica Pérez Fornos

**Affiliations:** ^1^Service of Otorhinolaryngology Head and Neck Surgery, Department of Clinical Neurosciences, Geneva University Hospitals, Geneva, Switzerland; ^2^Division of Balance Disorders, Department of ENT, Maastricht University Medical Centre, Maastricht, Netherlands; ^3^Faculty of Physics, Tomsk State University, Tomsk, Russia; ^4^Brandenburg University of Technology Cottbus—Senftenberg, Senftenberg, Germany

**Keywords:** bilateral vestibular loss, bilateral vestibulopathy, vestibular implant, video head impulse test, vestibulo-ocular reflex, electrical stimulation, cochlear implant

## Abstract

The purpose of this study was to evaluate whether it is possible to restore the high-frequency angular vestibulo-ocular reflex (aVOR) in patients suffering from a severe bilateral vestibulopathy (BV) and implanted with a vestibular implant prototype. Three patients (S1–3) participated in the study. They received a prototype vestibular implant with one to three electrode branches implanted in the proximity of the ampullary branches of the vestibular nerve. Five electrodes were available for electrical stimulation: one implanted in proximity of the left posterior ampullary nerve in S1, one in the left lateral and another one in the superior ampullary nerves in S2, and one in the right lateral and another one in the superior ampullary nerves in S3. The high-frequency aVOR was assessed using the video head impulse test (EyeSeeCam; EyeSeeTec, Munich, Germany), while motion-modulated electrical stimulation was delivered *via* one of the implanted vestibular electrodes at a time. aVOR gains were compared to control measurements obtained in the same patients when the device was not activated. In three out of the five tested electrodes the aVOR gain increased monotonically with increased stimulation strength when head impulses were delivered in the plane of the implanted canal. In these cases, gains ranging from 0.4 to values above 1 were measured. A “reversed” aVOR could also be generated when inversed stimulation paradigms were used. In most cases, the gain for excitatory head impulses was superior to that recorded for inhibitory head impulses, consistent with unilateral vestibular stimulation. Improvements of aVOR gain were generally accompanied by a concomitant decrease of corrective saccades, providing additional evidence of an effective aVOR. High inter-electrode and inter-subject variability were observed. These results, together with previous research, demonstrate that it is possible to restore the aVOR in a broad frequency range using motion-modulated electrical stimulation of the vestibular afferents. This provides additional encouraging evidence of the possibility of achieving a useful rehabilitation alternative for patients with BV in the near future.

## Introduction

Every day we are confronted to a variety of dynamic situations where precise head and body motion information are required to guarantee adequate function, safety, and well-being. The vestibular system is one of the main input channels mediating dynamic behavior, mainly through ocular and postural reflexes. One of the major functions of the vestibular system is the generation of compensatory eye movements during head motion to achieve gaze stabilization. This, for example, allows the perception of a stable visual environment during walking or running. Gaze stabilization is mainly achieved with the angular vestibulo-ocular reflex (aVOR), which is one of the key functions of the semicircular canals.

The high-frequency aVOR can be easily quantified by applying brisk, passive, and unpredictable head rotations (i.e., head impulses) in the plane of each semicircular canal while the tested subject fixates a visual target. In healthy subjects, a compensatory eye movement is generated in the opposite direction from that of the head impulse by the aVOR. By contrast, in cases of semicircular canal paresis, the aVOR is deficient and the affected subject needs to perform a compensatory saccade in order to maintain fixation on the target. The clinical observation of these compensatory saccades after head impulses has been used as a marker of semicircular canal dysfunction for several decades [head impulse test (HIT); (1)]. However, the diagnostic accuracy of this clinical evaluation, known as the HIT, remains limited since compensatory saccades can only be observed by the naked eye when they are “overt” (i.e., occurring once the head impulse has ended), while the relatively frequent “covert” saccades (i.e., occurring during the head impulse) remain undetected during clinical examinations (2). The video head impulse test (vHIT) is the technical evolution of the HIT, made possible by the development of high speed recording systems comprising high rate video cameras (3) and motion sensors (4, 5). vHIT systems allow the synchronous recording of head and eye movements, allowing side-specific, objective assessment of the aVOR allowing significantly improved diagnostic sensitivity (5). Not only can corrective saccades be accurately assessed but the aVOR gain (i.e., ratio of eye motion with respect to head motion) can also be computed, thereby allowing quantification of the severity of canal function loss. Today, the vHIT has become the gold standard in vestibular testing in the high-frequency domain (3–5).

The specificity of the vHIT also makes it an ideal candidate for the evaluation of the efficacy of rehabilitation interventions, in particular of vestibular implants. These are devices analogous to cochlear implants, designed to “artificially” restore loss of vestibular function using motion-modulated electrical currents delivered directly to the vestibular afferents. Current vestibular implants are designed to primarily restore semicircular canal function in patients suffering from bilateral vestibulopathy (BV) (6). Although often poorly acknowledged and underestimated, these patients are chronically and severely handicapped (7) and no effective treatment is available today.

Our group has investigated the feasibility of vestibular implants in humans for more than 10 years. We have developed special surgical procedures to allow access to the superior, posterior, and lateral ampullae (8) and ampullary nerves (9–11). The possibility of activating the aVOR pathway was verified, both in acute intra-operative settings as well as in 13 chronically implanted patients (12–14). Finally, we demonstrated that motion-modulated electrical stimulation of the lateral ampullary branch allowed to restore a close-to-normal VOR gain when using motion stimuli consisting of sinusoidal earth vertical (i.e., horizontal) rotations up to 2 Hz with a maximal angular velocity of 30°/s (15, 16). Nevertheless, head angular rotations during everyday activities typically involve higher angular velocities and frequencies in the three dimensional space, not only the horizontal plane. Investigating vHIT responses upon motion-modulated electrical stimulation appears thus as a fundamental extension of the assessment battery of artificial vestibular function. The purpose of this study was to fill this gap and evaluate the possibility of restoring the high-frequency aVOR measured by the vHIT using motion-modulated electrical stimulation of the vestibular nerve.

## Materials and Methods

### Patients and Device

Three patients fitted with a prototype vestibular implant were available for this study (Table 1). They were recruited at the Service of Otorhinolaryngology and Head and Neck Surgery at the Geneva University Hospitals according to strict criteria described in detail previously (14). Note that the inclusion criteria included a pathological vHIT response (e.g., gain <20%) for the six semicircular canals.

**Table 1:** Demographics, implantation, and experimental details of participating patients.

Patient	Sex	Etiology	Age (implant)	Implantation year	Surgical approach	Implanted side	Implanted electrodes	vHIT conditions	Baseline stimulation level (dynamic range) (μA)	Slopes linear transfer function (μA/°/s)
S1	M	Idiopathic	46	2008	EL	Left	PAN	RALP	300	−4, −2, 0, 2, 3, 4

S2	F	Traumatic	67	2013	IL	Left	PAN	Not tested	N/A	N/A
							SAN	LARP	400	−1, −0.5, 0. 0.5, 1, 2
							LAN	Horizontal	200	−0.5, 0, 0.5, 1, 2, 3

S3	M	Traumatic	53	2015	IL	Right	PAN	Not tested	N/A	N/A
							SAN	RALP	350	−3, −2, 0, 1, 2, 3
							LAN	Horizontal	400	−3, −2, 0, 2, 3

*PAN, posterior ampullary nerve; SAN, superior ampullary nerve; LAN, lateral ampullary nerve; EL, extralabyrinthine; IL, intralabyrinthine*.

Briefly, the vestibular implant prototype consisted of a modified cochlear implant (MED-EL, Innsbruck, Austria) where one to three electrodes were taken out of the cochlear array and put in separate branches. These vestibular branches were implanted in the vicinity of the ampullary nerves using the previously described intralabyrinthine (IL) or extralabyrinthine surgical techniques. Briefly, for the IL approach, milimetric fenestrations of the semicircular canals were performed. The electrodes were then manually inserted toward the ampulla. For the extralabyrinthine approach, the ampullary branches of the vestibular nerve were exposed without opening the labyrinth (8–11). The cochlear array was implanted using a regular round window approach. The electrode implanted near the posterior ampullary nerve (PAN) in patients S2 and S3 was not tested since in previous experiments we observed that these electrodes were not functional and did not evoke any vestibular responses. This is most likely due to fibrosis observed during the surgery, which precluded optimal electrode positioning. In both subjects the etiology was traumatic with the presence of an intraotic fracture crossing the ampulla of the posterior semicircular canal.

### Video Head Impulse Test

Video head impulse test was used to assess the high-frequency aVOR using the EyeSeeCam system (EyeSeeTec, Munich, Germany). This system comprises lightweight goggles that were fitted tightly to the patient’s head to reduce goggle slippage. The patient was seated 1.2 m in front of a fixation target at eye level. The system was calibrated with the patient alternating fixation on five dots, 8.5° apart, projected onto the wall in front of them. Head impulses were passive high-acceleration, small amplitude head rotations in the plane of the lateral semicircular canals (horizontal) as well as in the planes of the right anterior–left posterior (RALP) and left anterior–right posterior (LARP) semicircular canals (1). During head impulse testing, gaze was oriented according to the tested canal: straight ahead for horizontal impulses and 45° to the left or right for the RALP and LARP impulses. Eye movements were measured by video-oculography while head movements were recorded using integrated 6-degree-of-freedom inertial sensors (4). Eye and head movement data were synchronously sampled at a rate of 220 Hz. At least 12 valid head impulses were recorded in each experimental condition.

Video head impulse data were analyzed offline using custom MATLAB R2015b software (Mathworks, Natick, USA). In order to objectively evaluate the efficacy of the aVOR in the appropriate plane, only one electrode was stimulated at a time and only the angular head and eye velocity around the axis in the plane of the stimulated canal was taken into account. Head velocity exceeding 20°/s was considered as the start of the head impulse. The head impulse ended when eye velocity crossed 0 again. For each head impulse, the peak head velocity was recorded and the gain of the VOR was determined as the ratio of the median of eye and head angular velocity between 55 and 65 ms after the head impulse start. The maximum head velocity vs. gain vectors was smoothed using the robust LOWESS method to achieve an accurate representation of the correlation of both variables and minimize the effect of outliers (17, 18). The smoothing fraction was chosen to correspond to an interval of 50°/s in peak head velocity, similar to previous vHIT investigations (19).

### Electrical Stimulation

As already described in previous publications, to generate bidirectional eye movements (i.e., upward and downward when stimulating the vertical nerve branches; leftward and rightward when stimulating the horizontal nerve branch) using unilateral vestibular stimulation, it was necessary to first restore and maintain a *baseline activity* of the vestibular nerve (13–15). This was achieved by delivering a constant amplitude electrical stimulus (baseline stimulation). In this study, we chose a supraphysiological baseline stimulation profile consisting of trains of biphasic, charge-balanced pulses (200 μs/phase) presented at a rate of 400 pulses per second. These stimulation parameters were selected because they have proved to be particularly effective for activating the vestibular system in humans using our prototype devices. The amplitude of the baseline stimulation was set in the middle of the dynamic range measured for each patient (14). Once in the *adapted* state [i.e., after all symptoms related to the restitution of baseline activity of the vestibular nerve subsided; (13)], the amplitude of the baseline pulse train could be up- and down-modulated (amplitude modulation) to generate controlled aVOR responses. All stimulations were done in a pseudomonopolar configuration. The ground electrode was in the stimulator case which was located in the retroauricular region.

A regular cochlear implant processor (TEMPO+, MED-EL, Innsbruck, Austria) fitted with a customized transformation unit connected to the auxiliary input of the processor (20) was used to control the electrical stimulation currents delivered during the experiments. The head motion signal was captured with a three-axis gyroscope (LYPR540AH, STMicroelectronics, Geneva, Switzerland) fixed to the patient’s head using the head band of the vHIT device. This head motion signal was used to modulate the amplitude of the electrical stimulation delivered by the vestibular electrodes. To avoid slippage of the sensor, the tightly fixed head band was never touched during experiments. The hands of the examiner were positioned on the mastoid tips and the mandibles for horizontal impulses and on the vertex and the mandibules for LARP and RALP impulses. The temporomandibular joint was stabilized during tests by having patients bite tightly a wooden tongue depressor.

Head impulses were considered excitatory or inhibitory, depending on the implanted side and the direction of the impulse. For example, for patients implanted on the left ear (S1 and S2) a horizontal head rotation to the left was classified as an excitatory head impulse. A horizontal head rotation to the right corresponded to an inhibitory head impulse. Correspondingly, for patients implanted on the right ear (S3), a head impulse to the right was excitatory and a head impulse to the left was inhibitory. The same logic prevailed for the vertical canals. For the superior semicircular canals, a downward head impulse in their respective plane was considered excitatory, while an upward head impulse in the same plane was inhibitory. Finally, for the posterior semicircular canals, an upward head impulse in their respective plane was considered excitatory, while a downward head impulse in the same plane was inhibitory. This classification was based on the normal physiology of the healthy vestibular system (21, 22).

**Figure 1:**
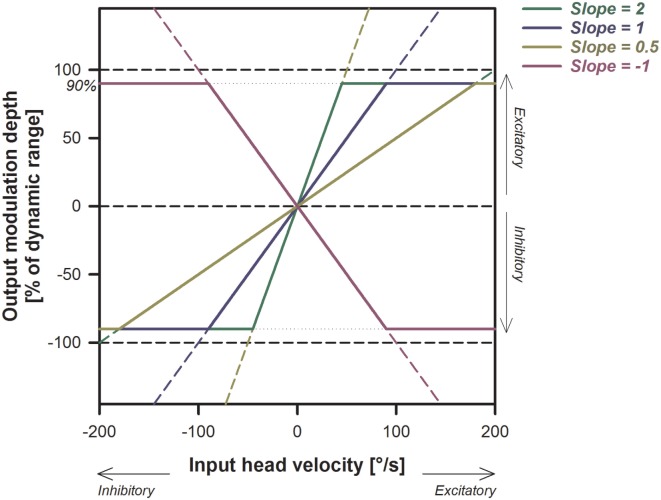
Illustration of the electrical stimulation paradigm and its expected effects on the stimulation output. Examples of different linear transfer functions with slopes ranging from −1 to +2 are presented. Note that the increase in the output (modulation strength) is steeper for larger slopes. Positive slopes (yellow, blue, and green solid lines) generate excitatory stimulation (up-modulation) for excitatory head movements and inhibitory stimulation (down-modulation) for inhibitory head movements. Negative slopes (pink solid line) have the opposite behavior.

We arbitrarily chose to implement simple linear transfer functions between measured yaw or pitch head velocity and electrical stimulation delivered *via* the electrodes implanted in the vicinity of the lateral, posterior, or superior ampullary nerves (respectively, LAN, PAN, and SAN). Transfer functions with different slopes (expressed in µA/°/s) were evaluated per subject and were defined based on the previously measured dynamic range and eye movement response characterized for each subject [see Table 1; (14, 23)]. Figure 1 shows examples of typical transfer functions used in these experiments. A higher slope of the linear transfer function implies that stronger modulation depths were generated for a given head velocity (i.e., higher electrical currents). Positive slopes resulted in excitatory electrical stimulation (up-modulation) for excitatory head impulses and in inhibitory stimulation (down-modulation) for inhibitory head impulses. Conversely, negative slopes delivered “reversed” motion information: inhibitory stimulation for excitatory head impulses and excitatory stimulation for inhibitory head impulses. Based on known physiology, we expected that positive slopes would generate a compensatory aVOR (eye movements in the opposite direction to the head), while negative slopes were expected to generate eye movements following the direction of head rotation (i.e., a “reversed” aVOR). Note that in this paradigm, a slope of 0 µA/°/s means that only constant amplitude baseline stimulation is delivered through the active electrode, but motion does not modulate the electrical signal. For safety reasons, maximum stimulation delivered was hard coded to be limited to 90% of the patient’s dynamic range, to avoid excessively high currents.

### Statistical Analyses

Correlations between variables were explored using Pearson product-moment correlation with a Bonferroni corrected significance level of 0.005 (0.05/10 to adjust for repeated testing). The influence of stimulation condition on smoothed aVOR gains was evaluated using two-way analysis of variance (ANOVA) with head impulse direction (excitatory/inhibitory) and stimulation condition (system OFF and slopes of the transfer functions) as analysis factors. For this test, we used a stringent significance level of 0.01 because the assumption of the homogeneity of variances could not always be verified. *Post hoc* comparisons were done with the Tukey HSD test with a significance level of 0.05. All statistical analyses were carried out with SigmaPlot version 13 (Systat Software, San Jose, CA, USA).

### Ethics

Patients gave their written informed consent to the study, which protocol was approved and carried out in accordance with the recommendations of the local ethics committee (Geneva University Hospitals NAC 11-080) and was designed in accordance with the declaration of Helsinki.

## Results

**Figure 2:**
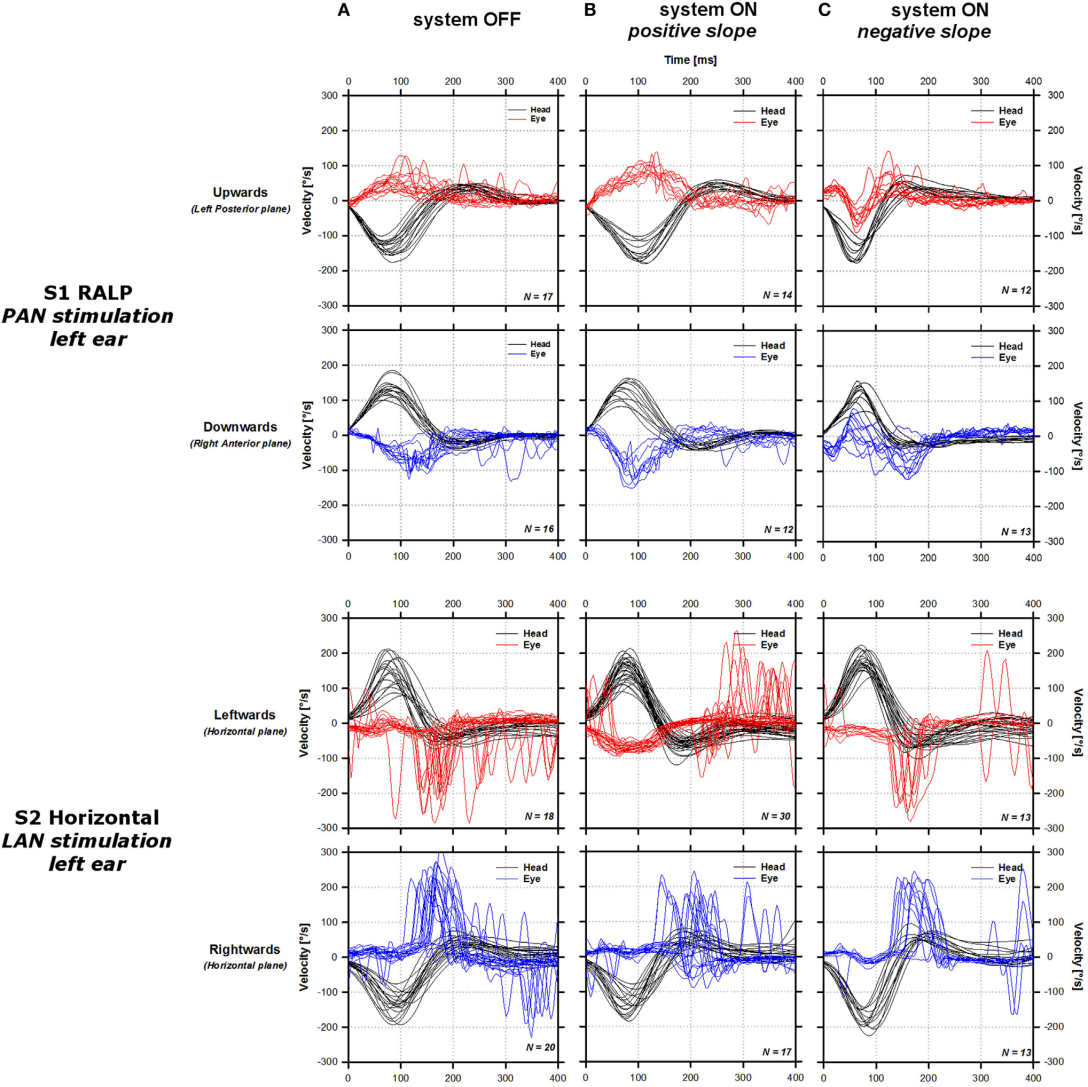

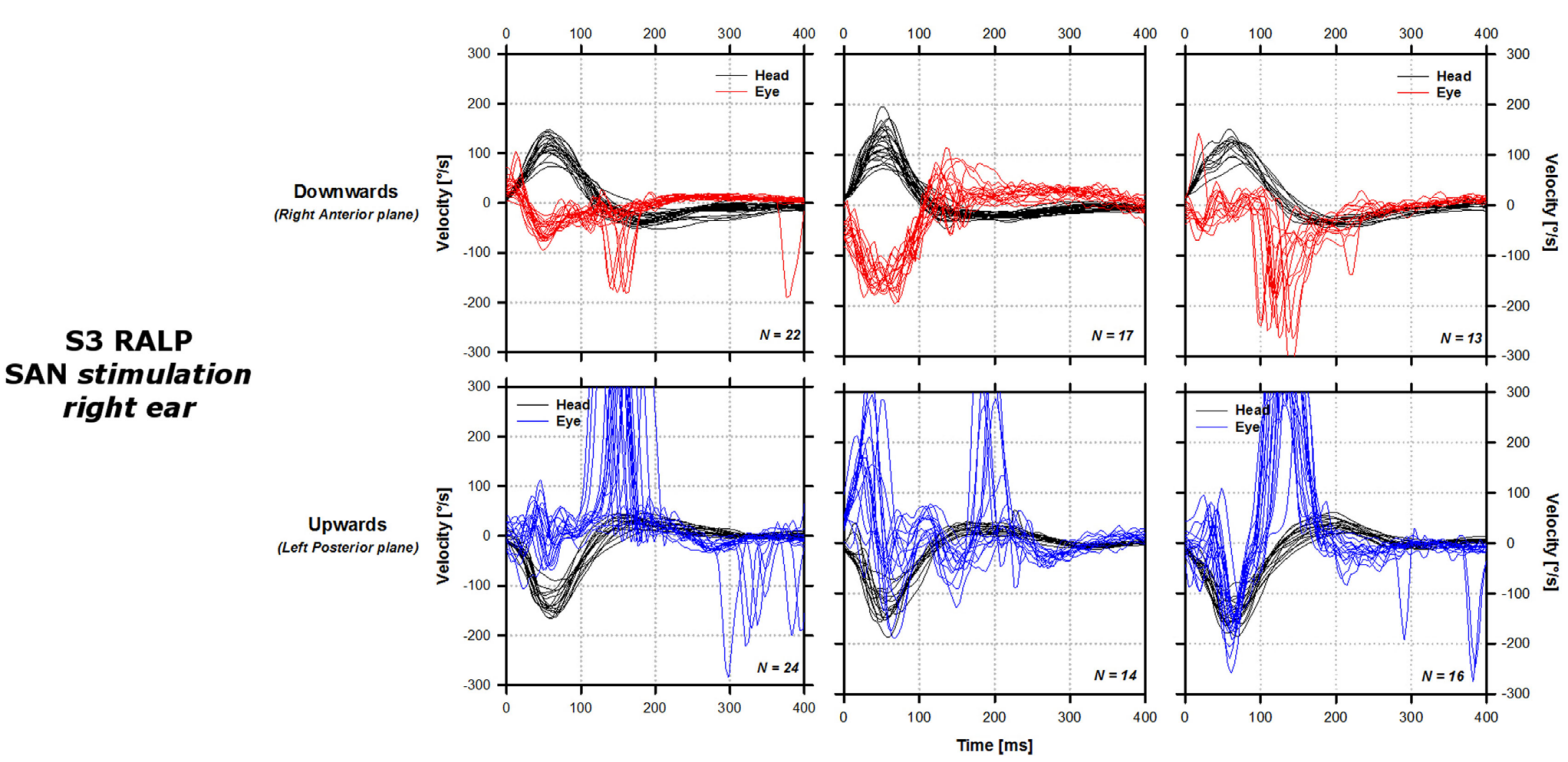
Sample video head impulse test responses of the three tested patients around one of the tested planes. For each patient [S1 right anterior–left posterior (RALP), upper lines; S2 horizontal, middle lines; S3 RALP, lower lines], the panels in the first column show data gathered without stimulation [system OFF condition **(A)**]. The panels in the second column show data gathered upon stimulation using a linear transfer function with a positive slope [system ON positive slope **(B)**; S1: 4 µA/°/s, S2: 3 µA/°/s, S3: 2 µA/°/s]. Panels in the third column show data gathered upon stimulation using a linear transfer function with a negative slope [system ON negative slope **(C)**; S1: −2 μA/°/s, S2: −0.5 μA/°/s, S3: −2 μA/°/s]. Solid black lines represent the cycle plots of the angular velocity of the head around the tested plane. Solid red and blue lines represent the cycle plots of the angular velocity of the eye in the plane of the tested canal for excitatory (red) and inhibitory impulses (blue). Positive values correspond to motion directed leftward in the horizontal plane, and motion directed downward in the vertical plane.

Representative examples of vHIT responses are presented in Figure 2 (S1 RALP, upper lines; S2 Horizontal, middle lines; S3 RALP, lower lines). In the three cases presented, the aVOR responses measured without activation of the vestibular implant (system OFF condition, first column in Figure 2) were deficient in both directions of the tested plane (excitatory: red solid lines; inhibitory: blue solid lines). Note that a low latency (<50 ms) artifact could be observed in the aVOR responses of S3. This rapid response is not likely an aVOR response, but presumably attributable to goggle slippage. Numerous saccades could be observed in S2 and S3, consistent with the deficiency of the aVOR. However in S1, corrective saccades were less frequent and of smaller amplitude.

Upon stimulation, the shape of the aVOR response changed. When positive transfer function slopes were used (second column in Figure 2), the shape of the aVOR improved for both excitatory and inhibitory head impulses in S1, but only for excitatory head impulses in S2 and S3. It was accompanied by a concomitant decrease in the frequency of compensatory saccades. Inverting the polarity of the slope of the linear transfer function resulted in an inversion of the aVOR response in S1 and S3, as well as an increase in the frequency and amplitude of compensatory saccades in S3, mainly for inhibitory head impulses. For S2, the response appeared similar to that observed in the system OFF condition. vHIT responses obtained upon stimulation of the SAN in S2 (LARP plane) and of the LAN in S3 (horizontal plane) are not shown because they were of similar shape than those recorded in the system OFF condition. We concluded that no aVOR could be successfully evoked using this two electrodes and therefore not considered them further in the analysis.

### aVOR Gain vs. Peak Head Angular Velocity

**Figure 3:**
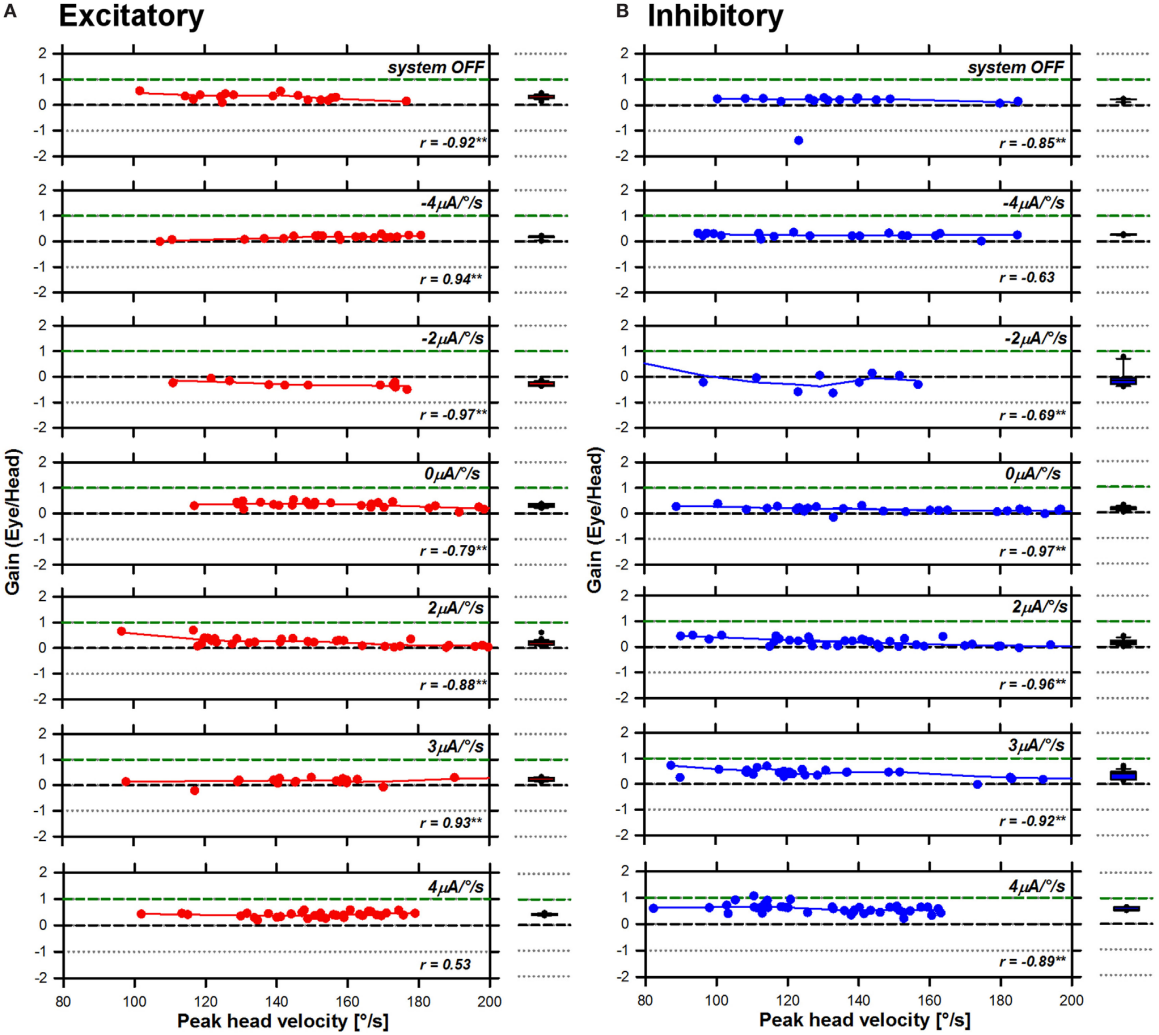
Angular vestibulo-ocular reflex (aVOR) gains recorded for S1 right anterior–left posterior in all experimental conditions. Panel **(A)** presents data gathered during excitatory head impulses (red plots), and panel **(B)** presents data gathered during inhibitory head impulses (blue plots). The line and scatter plots in the main panel present individual aVOR gains (scatter) as well as their corresponding smoothed (LOWESS, see Materials and Methods) values (lines) as a function of peak head angular velocity. The insets in the graph present the result of the statistical analysis for each condition (Pearson’s product-moment correlation coefficient; **p < 0.001—two-tailed). The box plots to the right present the median values, as well as the 25th–75th percentiles of the smoothed data pooled across head velocities. The error bars represent the 10th and 90th percentiles and the symbols present all outliers outside this range.

**Figure 4:**
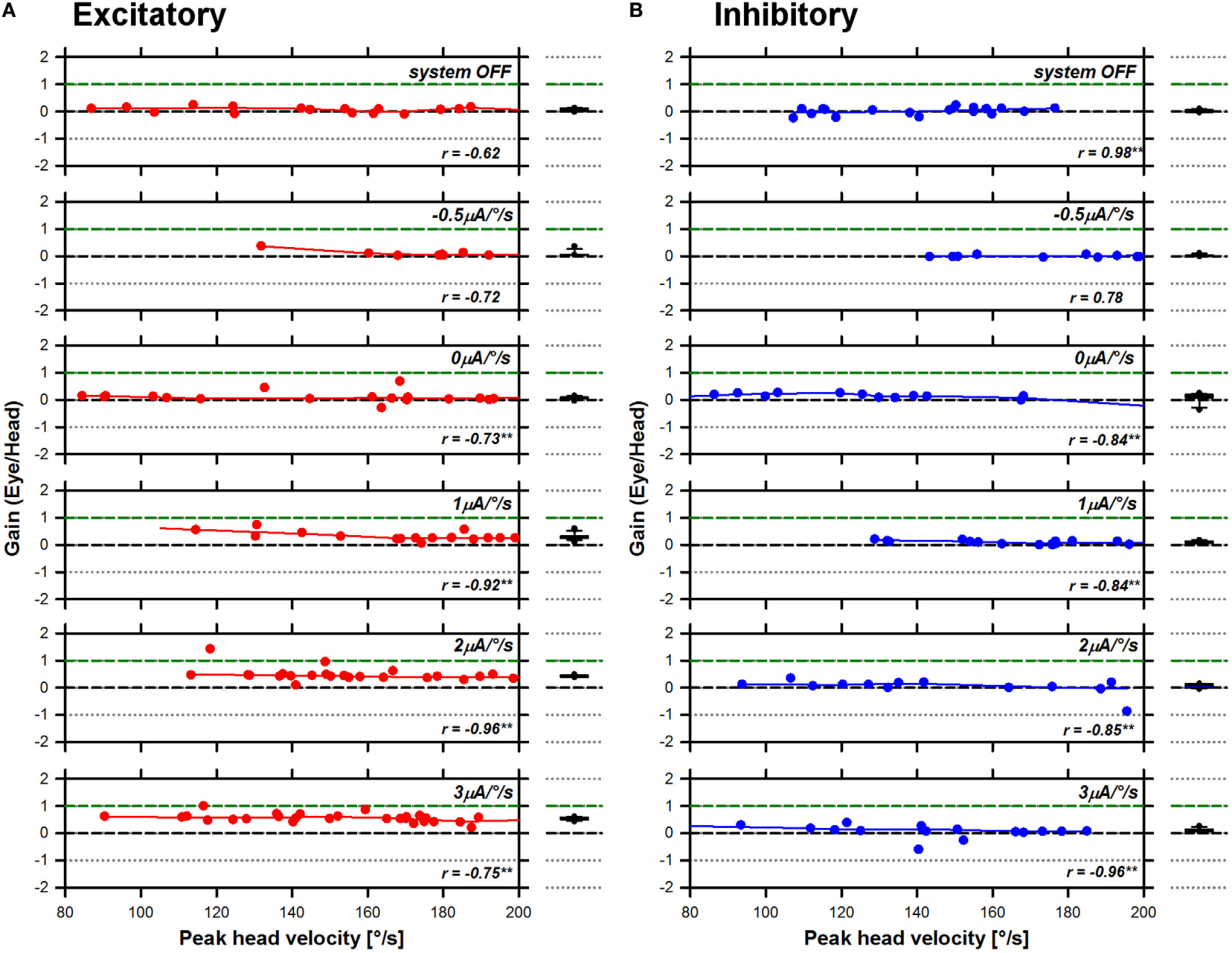
Angular vestibulo-ocular reflex (aVOR) gains recorded for S2 horizontal in all experimental conditions. Panel **(A)** presents data gathered during excitatory head impulses (red plots), and panel **(B)** presents data gathered during inhibitory head impulses (blue plots). The line and scatter plots in the main panel present individual aVOR gains (scatter) as well as their corresponding smoothed (LOWESS, see Materials and Methods) values (lines) as a function of peak head angular velocity. The insets in the graph present the result of the statistical analysis for each condition (Pearson’s product-moment correlation coefficient; **p < 0.001—two-tailed). The box plots to the right present the median values, as well as the 25th–75th percentiles of the smoothed data pooled across head velocities. The error bars represent the 10th and 90th percentiles and the symbols present all outliers outside this range.

**Figure 5:**
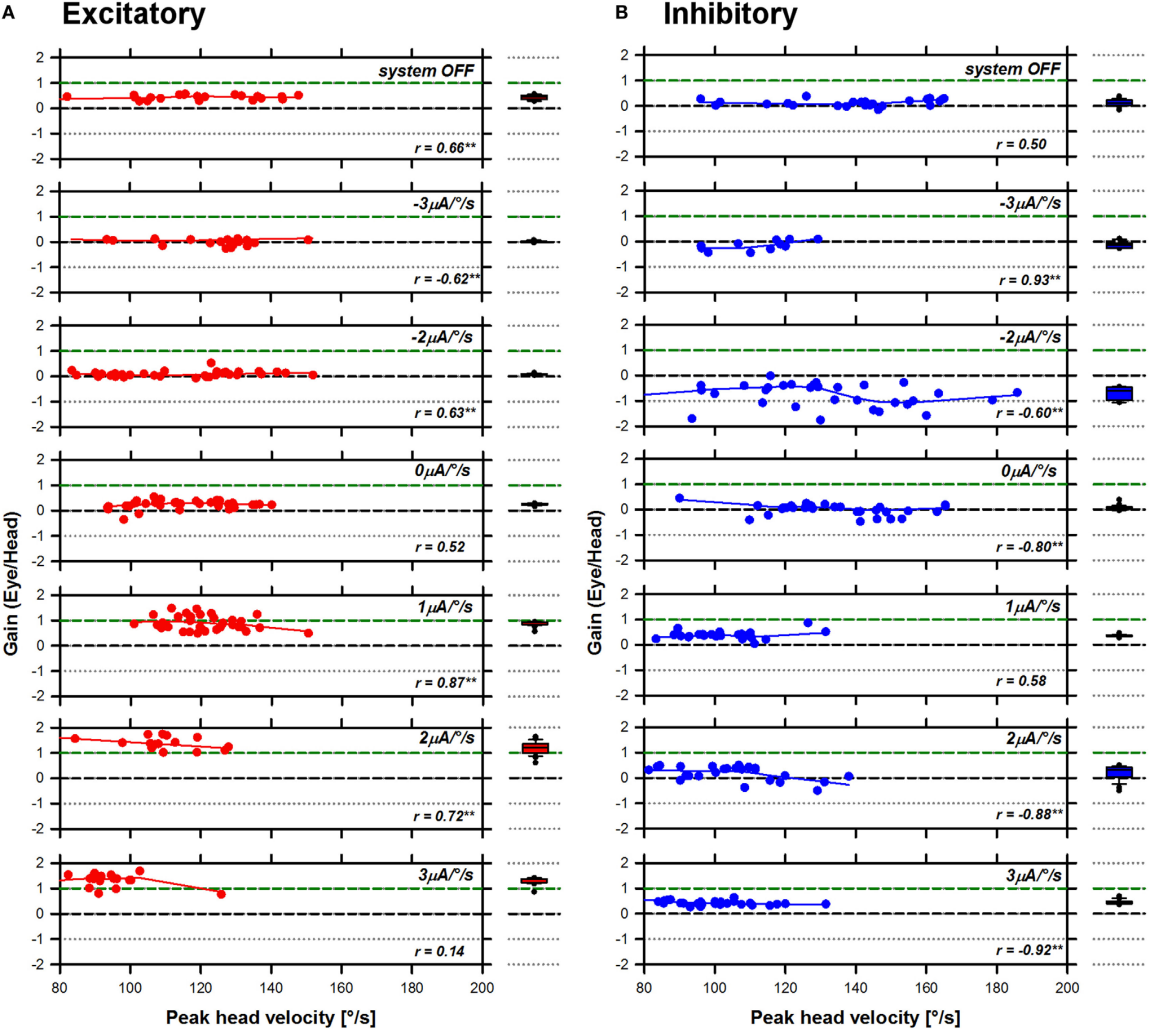
Angular vestibulo-ocular reflex (aVOR) gains recorded for S3 right anterior–left posterior in all experimental conditions. Panel **(A)** presents data gathered during excitatory head impulses (red plots), and panel **(B)** presents data gathered during inhibitory head impulses (blue plots). The line and scatter plots in the main panel present individual aVOR gains (scatter) as well as their corresponding smoothed (LOWESS, see Materials and Methods) values (lines) as a function of peak head angular velocity. The insets in the graph present the result of the statistical analysis for each condition (Pearson’s product-moment correlation coefficient; **p < 0.001—two-tailed). The box plots to the right present the median values, as well as the 25th–75th percentiles of the smoothed data pooled across head velocities. The error bars represent the 10th and 90th percentiles and the symbols present all outliers outside this range.

Previous studies conducted in healthy subjects demonstrated a significant influence of the motion stimulus profile (i.e., peak head angular velocity) on the aVOR response, particularly the gain (19). Therefore, as a first step in the analysis, we plotted the gain of the aVOR as a function of peak head angular velocity. These results are presented in Figure 3 (S1 RALP), Figure 4 (S2 horizontal), and Figure 5 (S3 RALP), for all experimental conditions. Overall, peak head angular velocities ranged between 80 and 200°/s, but were not uniformly distributed across conditions. The correlation between smoothed aVOR gains and peak head angular velocity was significant only in some cases (*p* < 0.001; see insets in Figures 3–5). However, even when present, the influence was small and the correlation was not systematic. Consequently, the median and the 25th–75th interquartile ranges of the pooled data appear to be a good representation of the behavior of the aVOR gains across the range of head velocities in each experimental condition. These are presented in the box plots to the right to the excitatory (A) and inhibitory (B) line and scatter plots in Figures 3–5 (red and blue plots, respectively).

### aVOR Gain As a Function of Stimulation Condition

**Figure 6:**
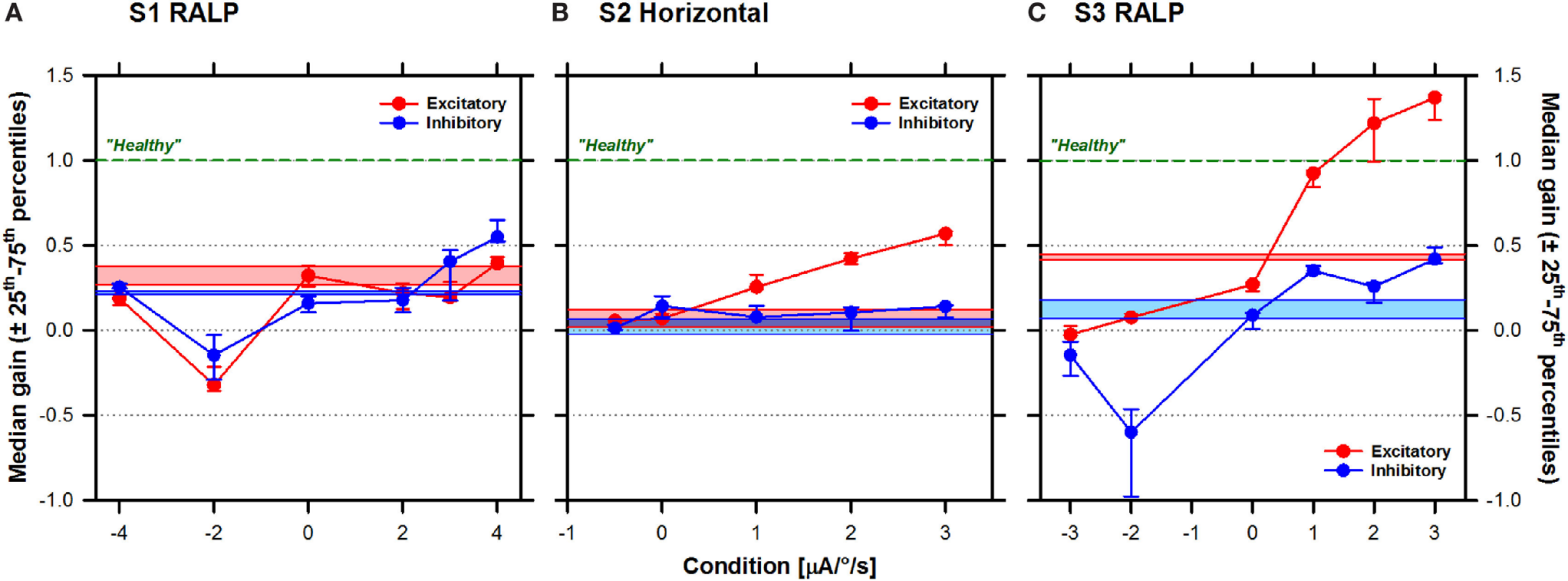
Median (±25th–75th percentiles) angular vestibulo-ocular reflex (aVOR) gains (vertical axis) for the three patients as a function of stimulation condition (horizontal axis): **(A)** S1 right anterior–left posterior (RALP), **(B)** S2 horizontal, and **(C)** S3 RALP. Note that a transfer function with a slope of 0 µA/°/s corresponds to constant amplitude electrical stimulation that is not modulated by motion (i.e., baseline stimulation only). Results for excitatory head impulses are plotted in red and results for inhibitory head impulses are plotted in blue. Results without electrical stimulation (system OFF) are presented as the colored solid bars in the graph. The green dotted line represents the theoretical aVOR gain of 1 for a normal subject with a “healthy” vestibular system.

The next step of our investigation was to explore the characteristics of the aVOR evoked in the different experimental conditions. Figure 6 presents the pooled aVOR gain data as a function of stimulation condition. The ANOVA analysis showed a statistically significant interaction effect of the direction of head impulses and the stimulation condition on aVOR gains, for the three subjects [S1: *F*_(6,379)_ = 27.69, *p* < 0.001; S2: *F*_(5,219)_ = 51.49, *p* < 0.001; S3: *F*_(6,370)_ = 99.06, *p* < 0.001]. Simple main effects *post hoc* analysis is presented in Table 2. In S1 (see Figure 6A), the median aVOR gain increased in the conditions where the slope of the transfer function increased (i.e., positive slope values) and tended to decrease for negative transfer function slopes. Although responses to both excitatory (red plots) and inhibitory (blue plots) head impulses showed similar trends, in this subject median aVOR gains were slightly, but significantly higher (*p* < 0.005) for inhibitory than for excitatory head impulses in most cases, reaching maximum values of 0.65 and 0.48, respectively. In most cases, these maximum aVOR gains were significantly higher than values obtained in all other conditions (*p* < 0.001). In S2 (Figure 6B), median aVOR gains for excitatory head impulses increased significantly (*p* < 0.001) with increasing positive transfer function slopes, reaching a maximum value of 0.69. Responses for inhibitory head impulses remained stable at a value near the one measured with the system OFF (see light colored bars in Figure 6), and only a small, significant (*p* < 0.001) increase was observed with the strongest stimulation condition (slope of 3 µA/°/s). The difference between aVOR gains measured for excitatory and inhibitory head impulses was statistically significant (*p* < 0.001) in all conditions where a positive transfer function slope was used. The effect of the stimulation condition was most striking in S3 (Figure 6C). In this subject, median gains reached values even above the normal “healthy” value of 1 (green dotted lines in Figure 6) in the two strongest conditions (2 and 3 µA/°/s). The effect of using transfer functions with negative slopes was also most striking in this subject, where the median aVOR gain decreased significantly, thereby reaching negative values for inhibitory head impulses. Finally, it can also be observed that responses for excitatory and inhibitory head impulses were also asymmetrical in S3 with statistically significant differences when comparing excitatory and inhibitory head impulses, at all levels (*p* < 0.002; see Table 2).

**Table 2:** *Post hoc* main effects (Tukey) analysis of angular vestibulo-ocular reflex gains for both factors included in the two-way analysis of variance analysis: stimulation condition (slope of the transfer function, μA/°/s), and head impulse direction (inhibitory/excitatory).

S1									
	**Stimulation conditions within excitatory**							**Excitatory vs. inhibitory**	

**Conditions**	**OFF**	**−4μA/**°**/s**	**−2μA/**°**/s**	**0μA/**°**/s**	**2μA/**°**/s**	**3μA/**°**/s**	**4μA/**°**/s**	**Conditions**	
**OFF**		***p* < 0.001**	***p* < 0.001**	*p* = 0.99	***p* = 0.003**	*p* = 0.010	*p* = 0.18	**OFF**	***p* < 0.001**
**−4μA/**°**/s**	*p* = 0.67		***p* < 0.001**	***p* < 0.001**	*p* = 0.28	*p* = 0.31	*p* = 0.48	**−4μA/**°**/s**	***p* < 0.001**
**−2μA/**°**/s**	***p* < 0.001**	***p* < 0.001**		***p* < 0.001**	***p* < 0.001**	***p* < 0.001**	***p* < 0.001**	**−2μA/**°**/s**	*p* = 0.005
**0μA/**°**/s**	*p* = 0.37	***p* < 0.001**	*p* = 0.03		***p* < 0.001**	***p* = 0.004**	***p* = 0.002**	**0μA/**°**/s**	***p* < 0.001**
**2μA/**°**/s**	*p* = 0.93	***p* < 0.001**	***p* < 0.001**	*p* = 0.86		*p* = 0.99	***p* < 0.001**	**2μA/**°**/s**	*p* = 0.07
**3μA/**°**/s**	***p* < 0.001**	***p* < 0.001**	***p* < 0.001**	***p* < 0.001**	***p* < 0.001**		***p* < 0.001**	**3μA/**°**/s**	***p* < 0.001**
**4μA/**°**/s**	***p* < 0.001**	***p* < 0.001**	***p* < 0.001**	***p* < 0.001**	***p* < 0.001**	***p* < 0.001**		**4μA/**°**/s**	***p* < 0.001**
	**Stimulation conditions within inhibitory**								

**S2**									

	**Stimulation conditions within excitatory**						**Excitatory vs. inhibitory**		

**Conditions**	**OFF**	**−0.5μA/**°**/s**	**0μA/**°**/s**	**1μA/**°**/s**	**2μA/**°**/s**	**3μA/**°**/s**	**Conditions**		
**OFF**		*p* = 0.99	*p* = 1.00	***p* < 0.001**	***p* < 0.001**	***p* < 0.001**	**OFF**	*p* = 0.07	
**−0.5μA/**°**/s**	*p* = 1.00		*p* = 0.99	***p* < 0.001**	***p* < 0.001**	***p* < 0.001**	**−0.5μA/**°**/s**	*p* = 0.04	
**0μA/**°**/s**	*p* = 0.05	*p* = 0.09		***p* < 0.001**	***p* < 0.001**	***p* < 0.001**	**0μA/**°**/s**	*p* = 0.30	
**1μA/**°**/s**	P = 0.45	*p* = 0.05	*p* = 1.00		***p* < 0.001**	***p* < 0.001**	**1μA/**°**/s**	***p* < 0.001**	
**2μA/**°**/s**	*p* = 0.44	*p* = 0.51	*p* = 0.96	*p* = 0.92		***p* < 0.001**	**2μA/**°**/s**	***p* < 0.001**	
**3μA/**°**/s**	***p* < 0.001**	***p* < 0.001**	*p* = 0.81	*p* = 0.85	*p* = 0.31		**3μA/**°**/s**	***p* < 0.001**	
	**Stimulation conditions within inhibitory**								

**S3**									

	**Stimulation conditions within excitatory**							**Excitatory vs. inhibitory**	
**Conditions**	**OFF**	**−3μA/**°**/s**	**−2μA/**°**/s**	**0μA/**°**/s**	**1μA/**°**/s**	**2μA/**°**/s**	**3μA/**°**/s**	**Conditions**	
**OFF**		***p* < 0.001**	***p* < 0.001**	***p* < 0.001**	***p* < 0.001**	***p* < 0.001**	***p* < 0.001**	**OFF**	***p* < 0.001**
**−3μA/**°**/s**	***p* < 0.001**		*p* = 0.24	***p* < 0.001**	***p* < 0.001**	***p* < 0.001**	***p* < 0.001**	**−3μA/**°**/s**	***p* < 0.001**
**−2μA/**°**/s**	***p* < 0.001**	***p* < 0.001**		***p* < 0.001**	***p* < 0.001**	***p* < 0.001**	***p* < 0.001**	**−2μA/**°**/s**	***p* = 0.002**
**0μA/**°**/s**	*p* = 0.66	***p* < 0.001**	***p* < 0.001**		***p* < 0.001**	***p* < 0.001**	***p* < 0.001**	**0μA/**°**/s**	***p* < 0.001**
**1μA/**°**/s**	***p* < 0.001**	***p* < 0.001**	***p* < 0.001**	***p* < 0.001**		***p* < 0.001**	***p* < 0.001**	**1μA/**°**/s**	***p* < 0.001**
**2μA/**°**/s**	*p* = 0.66	***p* < 0.001**	***p* < 0.001**	*p* = 0.01	***p* < 0.001**		***p* = 0.003**	**2μA/**°**/s**	***p* < 0.001**
**3μA/**°**/s**	***p* < 0.001**	***p* < 0.001**	***p* < 0.001**	***p* < 0.001**	*p* = 0.08	***p* < 0.001**		**3μA/**°**/s**	***p* < 0.001**
	**Stimulation conditions within inhibitory**								

*Significant values (*p* < 0.005) are highlighted in bold*.

## Discussion

The main goal of our study was to evaluate the efficacy of our vestibular implant prototype in restoring the high-frequency aVOR. Our results demonstrate that motion-modulated electrical stimulation of the ampullary branches of the vestibular nerve can be an effective means of restoring this reflex. The vHIT proved to be particularly suitable for this assessment, achieved for the first time in human patients suffering from BV.

We were successful in generating an “artificial” aVOR response upon stimulation of three out of the five tested electrodes: the PAN in S1, the LAN in S2, and the SAN in S3, for their corresponding planes. The aVOR gain increased as the strength of electrical stimulation increased, represented by an increase in the slope of the linear transfer function (see Figure 6). This is best illustrated by the results obtained in S3 (SAN stimulation, implanted on the right ear) where it was possible to normalize (>0.8) and even to obtain supranormal aVOR gains (>1) during excitatory head impulses (directed downward). A concomitant decrease of the number and amplitude of pathological corrective saccades generated during or after the head impulse was also observed (see Figure 2). When the slope of the transfer function was reversed (corresponding to a non-physiological situation where excitatory head rotations correspond to an inhibitory stimulus and *vice versa*), negative aVOR gains could be obtained in S1 and S3. This “reversed” aVOR, which is normally impossible to evoke with “natural” motion stimuli, also led to a concomitant increase in compensatory saccades in S3 (Figure 2). Altogether, these results clearly demonstrate that the vestibular implant can be successful in partially mimicking the physiology of the semicircular canals.

We observed significant asymmetry in the responses: except for S1, excitatory stimulation was more effective than inhibitory stimulation. This can be at least partially due to the fact that electrical stimulation was provided only unilaterally. Indeed, individual semicircular canals have an asymmetrical response for excitatory and inhibitory motion stimuli which was first described by Ewald (24) and has been thoroughly investigated since. For example, in squirrel monkeys it was observed that the resting discharge rate of the semicircular canal afferents (90 spikes/s) could be increased without saturation during excitatory head rotations. Similarly, the discharge rate during inhibitory head rotations decreases proportionally to angular acceleration (21). However in this latter case the nerve response saturates at 0 spikes/s. Therefore, a symmetrical aVOR requires bilateral stimulation, where the canal pairs (left lateral–right lateral; RALP; LARP) work in a push–pull configuration to achieve optimum responses in both directions of a given plane. This functional asymmetry is the cornerstone of the vHIT, and is used as a clinical marker for patients suffering from unilateral vestibular loss (25). It is thus not surprising that the vestibular implant, providing unilateral vestibular stimulation, generates asymmetrical responses. However, the fact that we were able to restore a considerable response, even if highly asymmetrical, holds great promise of being clinically relevant. Indeed, patients with a unilateral vestibular loss also show highly asymmetrical aVOR responses but have significantly higher quality of life scores than patients with a total bilateral vestibular loss (7). Furthermore, all three subjects of the present study were part of a previous investigation in which their visual acuity was assessed while walking in standardized condition with the vestibular implant (26). In all three subjects, the dynamic visual acuity could be significantly improved or even normalized when the vestibular implant was in the active mode. Although motion stimuli while walking have a predominant frequency of 2 Hz (27), which is below the frequency domain typically measured during the vHIT test, the positive functional outcomes of the dynamic visual acuity study are consistent with the aVOR gains obtained in the current study.

An important factor influencing the efficacy of the aVOR can be the choice of the stimulation paradigm. On the one hand, our stimulation strategy relies on the use of amplitude modulation to drive the target neural structures. This choice can be considered non-physiological, since vestibular afferents encode stimulus characteristics by increasing the discharge rate of the response, not its amplitude. We chose to use amplitude modulation for two main reasons. First, our vestibular implant prototype was built on the platform of a commercial cochlear implant. These devices implement amplitude and not rate modulation stimulation strategies. Second, our previous studies have demonstrated that amplitude modulation is an efficient way of activating the vestibular system, even more efficient than rate modulation in our particular setting involving distant stimulation of neural targets (13, 28, 29). On the other hand, we arbitrarily chose to implement a linear transfer function that avoids any theoretical assumptions regarding the relationship between the electrical stimulus and the vestibular response. Indeed, this linear transfer function has proven to be successful in restoring multimodal vestibular function (15, 16, 30). However, it does not mimic the non-linear response of the vestibular afferents (31, 32) nor the non-linear relationship between electrical stimulation and neural responses (33). Non-linear transfer functions (e.g., logarithmic, compressive) are currently implemented in other neuroprosthetic systems such as cochlear and retinal implants with good results. In the future, the use of non-linear transfer functions combined with different stimulation modes (e.g., amplitude-, rate- or co-modulation) might help to optimize the potential of the available dynamic range of each vestibular electrode (34). Bilateral implantation could also be considered. In this case, in theory, the full available dynamic range of each electrode could be devoted to the excitatory mode, with a potentially drastic improvement of the functional rehabilitation level.

An additional interesting finding of this study is the great variability observed across stimulation electrodes and across subjects. The first can be probably attributed to the misalignment of the aVOR response. Indeed, while we were successful in restoring the aVOR in 3 tested electrodes, electrical stimulation through the other 2 electrodes did not evoke any measurable response in the plane of the tested canal (SAN in S2 and LAN in S3). However, in both cases, the aVOR responses could be recorded in other planes (not shown). We observed that SAN stimulation in S2 evoked a predominantly horizontal response, and almost no response in the expected LARP plane. Conversely, LAN stimulation in S3 evoked an aVOR response in the RALP plane, similar (although smaller) to the one that would be expected for SAN stimulation. Based on these results, we assume that misalignment was due to current spread from one structure to the other, due to the anatomical proximity of the ampullae of the superior and lateral canals. This means that stimulation provided through one electrode or the other was probably stimulating both structures at the same time. In this case, we would only be able to record the “strongest” response coming from the structure with the lowest activation threshold. In the future, improved stimulation strategies and anatomically inspired electrode designs should help to improve the selectivity of stimulation and to reduce current spread.

The reason(s) underlying the large inter-subject variability is more difficult to elucidate. For example, in S1, inhibitory stimulation surprisingly generated slightly better responses than excitatory stimulation (see Figure 2). The etiology of the bilateral vestibular loss in S1 is unknown. The fact that the patient remembers being clumsy as a child and unable to perform well in sports hints on a possible congenital origin. It is thus difficult to hypothesize being on the development of gaze stabilization mechanisms in such a patient. Another example is S2 where inhibitory stimulation generated no clear aVOR responses, while in the other two subjects, inhibitory responses could be successfully recorded, especially for “reversed” stimulation with negative transfer function slopes. The responses observed for S3 were much higher than those recorded in the other two subjects. In light of these observations, the source of the high inter-subject variability appears difficult to determine at this point and in this small patient population. Position of the electrode (intra-/extralabyrinthine, distance to the neural target), etiology of the bilateral vestibular loss (i.e., integrity of the neural target), duration of the deficit, and age of onset of the bilateral vestibular loss are only some factors which could influence the response. It is worth highlighting that high inter-subject variability is also observed in other successful neuroprosthetic devices such as cochlear implants. Even in this mature field where relatively large patient cohorts are available, the fundamental reasons for the variability in outcomes across subjects remain difficult to explain [see, e.g., Ref. (35)].

Finally, a particularly interesting observation should be mentioned. Head impulses were delivered manually by an experienced examiner. Care was taken to cover the largest range of head peak angular velocities, which have been shown to have a small but significant effect on aVOR gain (19). For this purpose, real-time feedback of peak head angular velocity was displayed on a screen. Although not perfectly uniform across patients and conditions, the effect of the peak head angular velocity on the aVOR gain was limited and therefore the distribution was considered acceptable. However, it was striking to notice that in S3 the range of peak head velocities that could be achieved when the system was ON markedly decreased. For example, high peak head angular velocities above 140°/s could not be generated in most of these conditions. Previous studies have demonstrated that electrical stimulation of the ampullary branches of the vestibular nerve can activate the vestibulocollic/spinal pathways (30), resulting among others in a modulation of the neck muscle activity (36). Stiffening of the neck during head impulses, as part of the complex gaze stabilization mechanism mediated by the vestibular system, could thus explain the reduced peak head angular velocity observed. We hope that future research using the unprecedented flexibility of the vestibular implant stimulation capabilities should help shed more light on the complex interplay of the vestibular mechanisms in the perception, generation, and control of dynamic behavior.

In conclusion, this study together with our previous research demonstrates that motion-modulated electrical stimulation of the ampullary branches of the vestibular nerve can be successful in restoring the aVOR across a broad head frequency range. The clinical relevance of this “artificial” aVOR has also been investigated in studies where a significant improvement of gaze stabilization abilities was achieved in a laboratory controlled setting. All this evidence holds good promise of achieving a first real alternative for rehabilitating patients with a BV and warrants further research efforts and increased interest in the field.

## Ethics Statement

Patients gave their written informed consent to the study, which protocol was approved and carried out in accordance with the recommendations of the local ethics committee (Geneva University Hospitals NAC 11-080) and was designed in accordance with the declaration of Helsinki.

## Author Contributions

All authors participated in the design of the experimental protocol, analysis, and interpretation of the results. NG, AF, SC, and MR carried out the experiments. NG and RB performed the implantations. AF, MR, and SC prepared the figures with the help of other authors. NG and AF wrote the manuscript, and all authors contributed to its editing.

## Conflict of Interest Statement

The authors disclose that MED-EL (Innsbruck, Austria) has provided financial support to other research projects (not shown here) as well as travel expenses for scientific meetings. ES participated in the development of the EyeSeeCam system. He is general manager and a shareholder of EyeSeeTec GmbH.
